# Family Vaccination Context Predicts HPV Vaccine Uptake Among Medical Students

**DOI:** 10.3390/vaccines14070569

**Published:** 2026-06-28

**Authors:** Farah Harb, Shakhnozakhon Mukhtorova, Akarsh Lal, Laila Al-Jerdi, Morhaf Al Achkar, Radhika Gogoi

**Affiliations:** School of Medicine, Wayne State University, 540 E. Canfield St., Detroit, MI 48201, USA; s.mukhtorova@gwmail.gwu.edu (S.M.); akarshl@wayne.edu (A.L.); laila.al-jerdi@med.wayne.edu (L.A.-J.); alachkar@wayne.edu (M.A.A.); gogoir@karmanos.org (R.G.)

**Keywords:** HPV vaccination, vaccine uptake, family vaccination, medical students, cancer prevention, social context

## Abstract

**Background/Objectives:** Human papillomavirus (HPV) vaccination is an important cancer prevention strategy, yet uptake remains incomplete. Although provider recommendation is a key driver of HPV vaccination, less is known about how future physicians’ own vaccination behaviors are shaped. This study examined whether family vaccination context was associated with HPV vaccine uptake among medical students, with attention to implications for future vaccine counseling and prevention training. **Materials and Methods:** We conducted a cross-sectional analysis of survey data from 230 medical students at Wayne State University School of Medicine. The primary outcome was self-reported HPV vaccination status, dichotomized as fully vaccinated versus not fully vaccinated or unsure. Predictors included family vaccination context, gender, religious affiliation, age, year in medical school, prior HPV education, HPV knowledge, and HPV stigma. Family vaccination context was modeled as an ordinal predictor based on agreement that family members of age were vaccinated. HPV knowledge was modeled using a 22-item baseline pre-survey composite score. HPV stigma was modeled as a 2-item composite assessing perceived stigma associated with HPV and disclosure. Analyses included univariate and bivariate tests, followed by complete-case multivariable logistic regression. A sensitivity analysis restricted the outcome to vaccinated versus unvaccinated students, excluding those who were unsure. **Results:** Of 230 students, 189 (82.2%) reported being fully vaccinated against HPV. In bivariate analyses, family vaccination context, gender, and HPV knowledge were associated with vaccination status, whereas religion, age, year in medical school, prior HPV education, and HPV stigma were not. In the adjusted model (*n* = 165), family vaccination context remained the strongest independent correlate of HPV vaccination status (OR, 3.95; 95% CI, 2.11–7.42; *p* < 0.001). Female gender was also associated with higher odds of vaccination (OR, 3.80; 95% CI, 1.01–14.29; *p* = 0.048), as was higher HPV knowledge (OR, 1.26; 95% CI, 1.03–1.53; *p* = 0.024). HPV stigma showed a borderline inverse association with vaccination status (OR, 0.53; 95% CI, 0.26–1.07; *p* = 0.076). Religion, age, year in medical school, and prior HPV education were not statistically significant after adjustment. The family vaccination association remained robust in the vaccinated-versus-unvaccinated sensitivity analysis (OR, 7.70; 95% CI, 2.45–24.26; *p* < 0.001). Exploratory secondary analyses suggested that family vaccination context may also be associated with stronger intention to recommend HPV vaccination to future patients and greater intent to vaccinate among students who were not fully vaccinated or unsure, although these findings should be interpreted cautiously. **Conclusions:** Perceived family vaccination context was the strongest and most consistent correlate of self-reported HPV vaccine completion among medical students in this cross-sectional study. Gender and HPV knowledge were also associated with vaccination status in the primary adjusted model, although these estimates were less precise and less consistent across model specifications. Findings should be interpreted as associations rather than causal effects. HPV prevention training may benefit from addressing not only factual knowledge, but also trainees’ vaccination histories, perceived family and social norms, HPV-related stigma, and comfort discussing vaccination as part of preparation for future cancer prevention counseling.

## 1. Introduction

Human papillomavirus (HPV) is one of the most common sexually transmitted infections worldwide and a major contributor to cancer-related morbidity and mortality [1]. Persistent infection with high-risk HPV types causes nearly all cervical cancers and contributes substantially to oropharyngeal, anal, vulvar, vaginal, and penile cancers [2,3,4]. Although safe and effective vaccines are widely available, HPV vaccination coverage in the United States remains incomplete, making vaccine uptake a continuing cancer prevention priority [5,6].

Physicians play a central role in HPV vaccination decisions, and strong provider recommendation is consistently associated with higher vaccine initiation and completion [7,8]. As future physicians, medical students will be important participants in sustaining and expanding HPV prevention efforts [9]. Prior work has identified gaps in health professional students’ HPV-related knowledge, vaccine awareness, and willingness to recommend vaccination, and educational interventions have often focused on improving knowledge and preparedness [10]. Studies among college-aged populations further suggest that HPV vaccination intentions and uptake are shaped by knowledge, attitudes, social norms, and perceived barriers [11,12]. However, less attention has been paid to medical students’ own HPV vaccination status as a behavioral outcome in its own right. Although prior work has examined HPV knowledge, educational exposure, and willingness to recommend HPV vaccination among health professional students, less is known about the demographic, educational, cognitive, attitudinal, and family-context correlates of medical students’ own HPV vaccination status. In particular, few studies have examined whether medical students’ vaccination status is associated with family vaccination context, perceived HPV stigma, religious affiliation, gender, prior HPV education, and HPV knowledge within the same analytic framework. Understanding these correlates is important because medical students occupy a dual role as recipients of preventive care and future physicians who will be expected to recommend HPV vaccination as part of routine cancer prevention [13]. Therefore, the purpose of this study was to identify correlates of self-reported HPV vaccination status among medical students. Specifically, we examined whether family vaccination context, HPV knowledge, prior HPV education, HPV stigma, gender, age, year in medical school, and religious affiliation were associated with HPV vaccination status. Identifying these correlates may help inform medical education strategies that prepare future physicians to engage in HPV-related cancer prevention counseling.

## 2. Materials and Methods

### 2.1. Study Design and Participants

This study used a cross-sectional analysis of survey data collected from allopathic medical students at Wayne State University School of Medicine, an urban medical school in Detroit, MI, USA. Students across all four academic years were invited to participate via institutional email during the spring 2025 semester. The survey invitation was distributed to all four class email lists, representing approximately 1200 enrolled medical students, based on an estimated class size of approximately 300 students per year.

Participation was voluntary and anonymous. The first 200 respondents who completed the survey received a USD 5 Amazon gift card provided through Amazon.com, Inc. (Seattle, WA, USA). Data collection occurred from 3 March to 21 March 2025. The survey opened on 3 March 2025, and one email reminder was sent approximately one week after the survey opened. The survey was administered using *Qualtrics XM*. Qualtrics; 2025. Accessed [2025] https://www.qualtrics.com (accessed on 5 May 2026).

A total of 299 survey responses were received, of which 234 were complete. After analytic exclusions, the final analytic sample included 230 respondents, corresponding to an approximate analytic response rate of 19.2% based on the estimated eligible student population.

This study did not involve chemicals, reagents, devices, instruments, commercial cell lines, commercial samples, or biological materials. The study was reviewed by the Wayne State University Institutional Review Board and determined not to meet criteria for Human Participant Research on 15 August 2024. The IRB number was 2024 163.

### 2.2. Survey Instrument

Participants completed an electronic survey administered through Qualtrics. The instrument included demographic items, HPV-related knowledge items, vaccination items, and attitudinal questions. The survey was developed by the study team for this educational and cancer-prevention context and was informed by prior literature on HPV knowledge, vaccination, stigma, and recommendation intention. Items were reviewed for relevance to medical students and HPV prevention education before dissemination. The questionnaire/survey questions were developed by Akarsh Lal and Dr. Gogoi and reviewed together before approval for the official survey. The Qualtrics survey was developed by Akarsh Lal and Laila Al-Jerdi. Questions were designed to assess HPV knowledge and HPV attitudes pre and post intervention. Answer choices were presented in Likert-style format. Demographic data such as religion, year in school, age, and gender were also collected. Gender was assessed using the survey item, ‘What is your gender?’ Response options included Male, Female, Non-binary, Transgender male/man, Transgender female/woman, genderqueer/genderfluid, agender, other, and prefer not to say. The survey also included a later post-infographic section; however, for the present analysis, knowledge was conceptualized as general HPV knowledge rather than an intervention-change construct. Accordingly, post-infographic items were excluded from the analytic knowledge score, and only the baseline knowledge items were used. The family-vaccination item and the item assessing prior HPV coursework or extensive study outside medical school were drawn from the baseline attitudinal section of the survey. The full survey instrument is provided as Supplementary File S1.

### 2.3. Primary Outcome

The primary outcome was self-reported HPV vaccination status, based on the survey item asking whether respondents were currently fully vaccinated against HPV. For the primary analysis, vaccination status was dichotomized as fully vaccinated versus not fully vaccinated or unsure. This coding approach was selected because the analytic focus was confirmed completion of HPV vaccination. From a cancer-prevention and medical-education perspective, students who were unsure of their vaccination status may still represent a group for whom vaccination-status verification, counseling, or catch-up guidance is needed. However, because uncertainty about vaccination status may be conceptually distinct from confirmed non-vaccination, a sensitivity analysis was conducted in which the outcome was restricted to fully vaccinated versus not vaccinated only, excluding respondents who reported being unsure.

### 2.4. Exploratory Secondary Approach

Exploratory secondary outcomes included intention to recommend HPV vaccination to future patients, intention to recommend HPV vaccination to friends or family, and, among students who were not fully vaccinated or unsure of their vaccination status, intention to receive HPV vaccination in the next 3 months. Intention to recommend HPV vaccination to future patients was analyzed using a top-box approach, comparing students who strongly agreed with recommending HPV vaccination to future patients with all other responses. This approach was selected because the item showed a substantial ceiling effect and was intended to distinguish strong recommendation commitment from general endorsement. Intention to recommend HPV vaccination to friends or family was analyzed as an ordinal outcome because responses were more broadly distributed. Intent to vaccinate among students who were not fully vaccinated or unsure was examined descriptively and in parsimonious exploratory analyses because of the small analytic sample.

### 2.5. Predictors

Predictors were selected to reflect demographic, educational, cognitive, attitudinal, and social-context domains relevant to HPV vaccination behavior. Conceptually, these domains were organized using a Health Belief Model-informed perspective in which preventive behavior may be shaped by knowledge, perceived barriers, modifying factors, and cueing or normative influences. The Health Belief Model was used as an interpretive framework rather than a fully specified theoretical test, because not all core constructs, such as perceived susceptibility, perceived severity, perceived benefits, perceived barriers, cues to action, and self-efficacy, were directly measured. The primary predictors evaluated were family vaccination context, gender, religious affiliation, age, year in medical school, prior HPV education, HPV knowledge, and HPV stigma.

Family vaccination context was treated as the primary independent variable of interest. Family vaccination context was derived from the baseline item asking respondents whether family members of age were vaccinated. This variable was modeled as an ordinal predictor ranging from strongly disagree to strongly agree. ‘Not sure’ responses were treated as missing rather than recoded to the midpoint. Because this measure was based on a single self-reported item, it was interpreted as a perceived family-level vaccination context rather than an objective measure of verified relatives’ HPV vaccination status. It may reflect actual family vaccination behavior, perceived family norms, family communication about vaccination, or the salience of HPV vaccination within the respondent’s family environment.

Gender was assessed using the survey item, “What is your gender?” Response options included Male, Female, Non-binary, Transgender male/man, Transgender female/woman, genderqueer/genderfluid, agender, other, and prefer not to say. For regression models, gender was analyzed as a binary indicator comparing respondents who selected Female with respondents who selected Male because other categories had sparse cell sizes.

Religion was collapsed into analytically broader categories to reduce sparsity: Christianity, Islam, no religion/atheist, and other religion. Religion was included as a contextual covariate because prior research has suggested that religious and cultural context may shape vaccine attitudes and decision-making. Prefer-not-to-say and missing responses were excluded from complete-case models.

Age was coded as an ordered variable based on the survey’s age ranges. Year in medical school was coded as an ordered variable from M1 to M4. Prior HPV education was coded as a binary indicator reflecting any prior HPV-related education. This variable combined the baseline item on formal HPV instruction in medical school with the item assessing prior HPV coursework or extensive study outside medical school. Respondents were coded as having prior HPV education if they reported either source of prior exposure.

### 2.6. HPV Knowledge Score

HPV knowledge was operationalized as a baseline-only composite score using the pre-survey knowledge items. Each item was scored dichotomously, with correct responses coded as 1 and incorrect or unsure responses coded as 0. Because the analytic goal was to capture general HPV knowledge rather than intervention-related change, post-infographic items were excluded. One item assessing whether two doses are recommended for immunocompromised persons was excluded from the composite because it functioned as a near-floor item and reflected ambiguity in HPV vaccine dosing guidance rather than general HPV knowledge. The resulting 22-item composite included items assessing HPV biology, transmission, HPV-associated cancers, oropharyngeal cancer, interpretation of cancer-trend information, and vaccine-related knowledge. The composite had acceptable internal consistency, with KR-20 reliability of 0.81. Higher scores indicated greater HPV knowledge.

### 2.7. HPV Stigma Score

HPV stigma was operationalized as a 2-item composite using baseline attitudinal items assessing whether HPV carries stigma similar to other sexually transmitted infections and whether there is stigma associated with disclosing an HPV diagnosis to family or friends. Items were scored from 1, strongly disagree, to 5, strongly agree, with not sure responses treated as missing. The composite was computed as the mean of available items. Internal consistency was acceptable, with Cronbach α = 0.71. Higher scores reflected greater perceived HPV-related stigma.

### 2.8. Statistical Analysis

We first conducted univariate analyses to summarize sample characteristics, missingness, outcome distribution, and the distribution of key predictors. Frequencies and percentages were used for categorical variables and means with standard deviations and medians with interquartile ranges (IQRs) were used for continuous or ordinal summary measures as appropriate. Observed ranges were reported descriptively when useful for scale interpretation. We then conducted bivariate analyses comparing HPV vaccination status across predictors. Categorical predictors were compared using chi-square tests, with sparse categories excluded from the test when necessary and retained descriptively. Ordinal predictors, including age category, medical school year, and family vaccination context, were compared using Mann–Whitney U tests. HPV knowledge and HPV stigma scores were also compared across vaccination groups using Mann–Whitney U tests because distributions were not assumed to be normal.

The primary adjusted analysis used complete-case binary logistic regression to estimate associations between predictors and HPV vaccination status. The main model included family vaccination context, gender, collapsed religion, age, year in medical school, prior HPV education, HPV knowledge score, and HPV stigma. Complete-case analysis was used because several covariates contained missing or analytically excluded responses, particularly ‘not sure’ responses for the family vaccination item and sparse demographic categories. This approach allowed a consistent analytic sample across predictors but reduced the adjusted analytic sample from 230 to 165 respondents. Multicollinearity among predictors included in the adjusted regression model was assessed using tolerance statistics and variance inflation factors. No evidence of problematic multicollinearity was observed. Variance inflation factor values ranged from 1.02 to 1.41, and tolerance values ranged from 0.711 to 0.985, indicating that predictors in the adjusted model were not highly collinear. A sensitivity binary logistic regression was conducted restricting the outcome to respondents who answered yes or no to HPV vaccination status, excluding those who were unsure. Because only a small number of unvaccinated respondents remained in that model, religion was collapsed to a binary Christian versus non-Christian indicator to improve model stability.

Exploratory secondary analyses examined associations between family vaccination context and intention-related outcomes. For intention to recommend HPV vaccination to future patients, a top-box binary logistic regression model compared respondents who strongly agreed with recommending HPV vaccination to future patients with all other respondents. Intention to recommend HPV vaccination to friends or family was analyzed using ordinal logistic regression, with higher values indicating stronger recommendation intention. Among students who were not fully vaccinated or unsure of their HPV vaccination status, intention to receive HPV vaccination in the next 3 months was examined descriptively and with parsimonious exploratory logistic regression models. Parsimonious models were limited to family vaccination context as the primary predictor, with personal HPV vaccination status included only when applicable, to avoid overfitting given the small analytic sample and sparse response patterns. Statistical significance was defined as a 2-sided *p* value less than 0.05. Analyses were conducted using SPSS. version 30 (IBM Corp., Armonk, NY, USA).

## 3. Results

### 3.1. Study Population

A total of 230 medical students were included in the final analytic sample. Overall, 189 students (82.2%) reported being fully vaccinated against HPV, 17 (7.4%) reported not being fully vaccinated, and 24 (10.4%) were unsure of their vaccination status. Participants represented all 4 class years. Most students were aged 23 to 26 years. Religious affiliation was heterogeneous, with Christianity reported most frequently, followed by no religion/atheism. Most respondents identified as female. A small number of respondents reported non-binary, transgender, or prefer-not-to-say gender identities and were retained in descriptive summaries but excluded from gender-adjusted regression models because of sparse cell sizes. Under the combined exposure definition, most respondents were classified as having prior HPV education. The 22-item HPV knowledge score demonstrated a relatively high overall level of knowledge in the sample. The 2-item HPV stigma composite showed moderate perceived stigma overall (Table 1).

### 3.2. Univariate Findings

Missingness was low for most variables, although the family-vaccination item had a substantial proportion of “not sure” responses that were treated as missing in regression analyses. HPV vaccination status itself had no missingness. The 22-item HPV knowledge score had a mean of 16.48 (SD, 4.13), median [IQR] of 18 [15, 19], and observed range of 1 to 22, with acceptable internal consistency (KR-20 = 0.81). The HPV stigma composite had a mean of 3.45 (SD, 0.97), median [IQR] of 3.50 [3.00, 4.00], and acceptable internal consistency (Cronbach α = 0.71).

### 3.3. Bivariate Associations

In bivariate analyses, family vaccination context, gender, and HPV knowledge were associated with HPV vaccination status, whereas religion, age, year in medical school, prior HPV education, and HPV stigma were not. Students who were fully vaccinated reported stronger agreement that family members of age were vaccinated than did students in the not-vaccinated-or-unsure group. Fully vaccinated students also had higher HPV knowledge scores. Respondents who selected Female reported higher vaccination prevalence than respondents who selected Male (Table 2).

### 3.4. Multivariable Model

In the primary multivariable logistic regression model, estimated using complete-case analysis (*n* = 165), family vaccination context remained the strongest independent correlate of HPV vaccination status. Each 1-step increase in family vaccination context was associated with nearly fourfold higher odds of being fully vaccinated (OR, 3.95; 95% CI, 2.11–7.42; *p* < 0.001). Selecting Female was also associated with higher adjusted odds of HPV vaccination compared with selecting Male (OR, 3.80; 95% CI, 1.01–14.29; *p* = 0.048), although the confidence interval was wide and the estimate should be interpreted cautiously given sparse cells. Estimates for religious-affiliation categories were imprecise because of small cell counts in some groups. Higher HPV knowledge was associated with higher odds of vaccination (OR, 1.26; 95% CI, 1.03–1.53; *p* = 0.024). HPV stigma showed a borderline inverse association with vaccination status (OR, 0.53; 95% CI, 0.26–1.07; *p* = 0.076). Religion, age, year in medical school, and prior HPV education were not statistically significant after adjustment. Because the number of respondents who were not fully vaccinated or unsure was limited relative to the number of model covariates, some estimates may be affected by reduced precision and model instability, particularly for predictors with sparse cells. Similar concerns have been described in the logistic regression literature when outcome events are limited relative to the number of predictors included in a model [14,15]. The primary adjusted model had a McFadden pseudo-R^2^ of 0.368 and an overall likelihood ratio test *p* value less than 0.001. See Table 3.

In the sensitivity analysis restricting the outcome to respondents who answered yes or no to HPV vaccination status, excluding those who were unsure, the association between family vaccination context and HPV vaccination remained robust. Each 1-step increase in family vaccination context was associated with higher odds of being fully vaccinated (OR, 7.70; 95% CI, 2.45–24.26; *p* < 0.001). Gender, HPV knowledge, prior HPV education, religion, age, and medical school year were not statistically significant in this restricted model. HPV stigma showed a borderline inverse association (OR, 0.22; 95% CI, 0.05–1.05; *p* = 0.057), but this finding should be interpreted cautiously because of the small number of unvaccinated respondents.

### 3.5. Exploratory Secondary Outcomes

Intention to recommend HPV vaccination to future patients showed a substantial ceiling effect, with most respondents endorsing agreement or strong agreement. In the adjusted top-box model, stronger family vaccination context was associated with higher odds of strongly agreeing that one would recommend HPV vaccination to future patients (OR, 2.23; 95% CI, 1.37–3.63; *p* = 0.001), whereas personal HPV vaccination status was not statistically significant after adjustment. In analyses of intention to recommend HPV vaccination to friends or family, family vaccination context was also positively associated with stronger recommendation intention. Among students who were not fully vaccinated or unsure of their vaccination status, stronger family vaccination context was associated with greater intent to receive HPV vaccination in the next 3 months. Because these analyses were exploratory and, for the intent-to-vaccinate subgroup, based on a small analytic sample, findings should be interpreted cautiously.

## 4. Discussion

This study found that perceived family vaccination context was the strongest and most consistent correlate of self-reported HPV vaccine completion among medical students. Students who more strongly agreed that family members of eligible age were vaccinated had higher odds of reporting that they themselves were fully vaccinated. This association remained statistically significant in both the primary adjusted model and the sensitivity analysis that excluded respondents who were unsure of their vaccination status. Female gender and higher HPV knowledge were also associated with vaccination status in the primary adjusted model, although these associations were less consistent across model specifications and should be interpreted cautiously given wide confidence intervals and sparse outcome categories.

These findings should be interpreted as associations rather than evidence of causal mechanisms. Because the study was cross-sectional, it cannot determine whether perceived family vaccination context influenced students’ HPV vaccination behavior, whether vaccinated students were more aware of family members’ vaccination status, or whether both reflected broader social, family, healthcare-access, or preventive-care factors. Nevertheless, the findings are consistent with prior HPV vaccination literature showing that vaccine decisions are shaped not only by factual knowledge, but also by provider recommendation, communication quality, perceived norms, and interpersonal context [7,8,10,11,12,13]. Although family vaccination context has been less frequently examined among medical students specifically, studies among adolescents, college students, and young adults have consistently demonstrated that family attitudes, parental vaccination behaviors, and perceived social norms are associated with HPV vaccine uptake and intention [11,12]. Our findings extend this literature by suggesting that these influences may remain relevant even among medically trained populations.

The finding related to perceived family vaccination context is particularly important because medical students are often assumed to make preventive health decisions primarily on the basis of biomedical knowledge or formal education. In this sample, HPV knowledge was associated with vaccination status in the primary adjusted model, but perceived family vaccination context showed the most robust association across analyses. This pattern suggests that even among medically trained populations, HPV vaccine uptake may be linked to perceived family-level norms or vaccination environments. However, because family vaccination context was measured using a single self-reported item, it should not be interpreted as verified family vaccination status. Instead, it may reflect respondents’ perceptions of whether HPV vaccination is known, discussed, normalized, or salient within their family environment.

The observed gender association also warrants cautious interpretation. Respondents who selected Female had higher adjusted odds of reporting HPV vaccination than respondents who selected Male, but the estimate was imprecise and was not statistically significant in the yes-versus-no sensitivity analysis. This pattern may reflect the historical gendering of HPV vaccination, including earlier public messaging that emphasized cervical cancer prevention and vaccination among girls and young women. This pattern is broadly consistent with prior studies reporting higher HPV vaccine uptake among women than men [11,12], particularly among cohorts who became age-eligible during periods when HPV vaccination campaigns primarily emphasized cervical cancer prevention. Although HPV vaccination is now recommended across genders and prevents multiple HPV-associated cancers, including cervical, oropharyngeal, anal, vulvar, vaginal, and penile cancers, earlier gendered framing may continue to shape vaccination histories among young adults [1,2,3,4,5,6]. Future studies with larger and more gender-diverse samples are needed to examine this pattern more precisely.

Religious affiliation was not independently associated with HPV vaccination status in adjusted models. This finding should be interpreted cautiously because some religious-affiliation categories were small, resulting in imprecise estimates. The absence of a statistically significant religion-specific association in this sample should not be interpreted as evidence that religion or culture is irrelevant to HPV vaccination decisions. Rather, it indicates that, within this sample and model specification, religion was not independently associated with vaccination status after accounting for family vaccination context, gender, HPV knowledge, stigma, and other covariates.

HPV stigma showed a borderline inverse association with vaccination status, but this finding should also be interpreted cautiously. The stigma measure was based on two items, and the number of students who were unvaccinated or unsure was small. The direction of the association is consistent with the possibility that stigma around HPV, sexual health, or disclosure may be relevant to vaccination behavior, but the present study cannot determine whether stigma influences vaccination decisions directly. Although evidence is mixed, prior studies have reported that HPV-related stigma, embarrassment, concerns about sexual health disclosure, and anticipated social judgment may reduce willingness to discuss HPV vaccination or seek preventive services [16,17,18]. The direction of association observed in our study is consistent with this broader literature, although the present findings were not statistically significant and should be interpreted cautiously. Future research should use fuller validated measures of HPV stigma and examine whether stigma operates through perceived norms, anticipated judgment, communication comfort, or willingness to seek vaccination.

The Health Belief Model provides a useful interpretive framework for these findings, but the present study should not be understood as a full test of the model. Not all core HBM constructs, including perceived susceptibility, perceived severity, perceived benefits, perceived barriers, cues to action, and self-efficacy, were directly measured [19]. Accordingly, the findings are best described as HBM-informed rather than confirmatory of the model. Within this cautious interpretation, family vaccination context may represent part of the social or cueing environment surrounding HPV vaccination, whereas HPV knowledge may reflect one cognitive component of vaccine decision-making. Broader normative frameworks, including the Theory of Planned Behavior, may also be relevant for future research that directly measures perceived norms, attitudes, intentions, and self-efficacy [20]. In addition, a possible explanation for the lack of independent associations for religion, age, year in medical school, and prior HPV education is the relatively high baseline educational background and medical training of the sample. Because participants were medical students, HPV-related knowledge, exposure to preventive health concepts, and acceptance of vaccination may have been higher and less variable than in general university or community samples. This restricted variability may have reduced the ability to detect associations for factors that are often more influential in broader KAP studies of HPV vaccination. Accordingly, the absence of statistically significant associations for these variables should be interpreted cautiously and should not be taken to mean that these factors are irrelevant in other populations.

The findings also have implications for medical education, although these implications should be framed as hypotheses for future intervention development rather than direct evidence of downstream provider behavior. Prior research has shown that provider recommendation and communication quality are important predictors of HPV vaccine uptake in patient populations, and medical students are future providers who will be expected to counsel patients about HPV vaccination [7,8,9,10]. The present study suggests that trainees’ own vaccination histories, perceived family norms, and comfort discussing HPV may be relevant topics for HPV prevention training. However, this study did not directly observe future counseling behavior, recommendation quality, or patient outcomes. Future studies should examine whether trainees’ personal vaccination status and perceived family vaccination context are associated with later communication confidence, recommendation practices, or patient-facing prevention behaviors.

Exploratory secondary analyses suggested that family vaccination context may be associated with intention-related outcomes, including stronger commitment to recommend HPV vaccination to future patients and greater intent to vaccinate among students who were not fully vaccinated or unsure. These findings should be interpreted cautiously because the analyses were exploratory, some outcomes showed ceiling effects, and the intent-to-vaccinate subgroup was small. They are best viewed as preliminary signals that require confirmation in larger samples with stronger measurement of recommendation intention, communication confidence, and future provider behavior.

This study has several limitations. It was conducted at a single institution, which may limit generalizability. Because participation was voluntary and the sample was drawn from one medical school, the findings may be affected by selection bias and may not represent all medical students. HPV vaccination status and family vaccination context were self-reported and were not verified through medical records or family reports, introducing potential recall and perception bias. The family vaccination context measure was based on a single survey item and may reflect perceived family norms, communication, or salience rather than objective vaccination status among relatives. The cross-sectional design precludes causal inference, and all findings should be interpreted as associations. For example, greater HPV knowledge may contribute to vaccine uptake, but students who have been vaccinated may also be more likely to recall, seek out, or receive HPV-related information through healthcare encounters. Similar limitations have been acknowledged in prior cross-sectional studies examining HPV vaccination determinants among college and healthcare trainee populations.

Model stability was also limited by the small number of students who were not fully vaccinated or unsure of their vaccination status relative to the number of covariates included in the adjusted model. This limitation is reflected in wide confidence intervals for several estimates, particularly gender, religion, and prior HPV education. The complete-case adjusted analysis included 165 of 230 respondents, so missing data and analytic exclusions may have affected the precision and robustness of adjusted estimates. If excluded respondents differed systematically from included respondents, selection bias may have influenced the results. Although the sensitivity analysis excluding unsure respondents supported the family vaccination finding, the small number of confirmed unvaccinated respondents limited inference in that model. A priori sample size calculation was performed because this study used available survey responses from a school-wide educational survey. Therefore, the analyses should be interpreted as exploratory and hypothesis-generating, particularly given the small number of respondents who were not fully vaccinated or unsure of their vaccination status.

Additional limitations include the grouping of unsure respondents with those not fully vaccinated in the primary analysis, which may obscure meaningful differences between uncertainty and confirmed non-vaccination. This choice was made to focus on confirmed vaccine completion and was evaluated through a yes-versus-no sensitivity analysis. Gender-diverse respondents were retained in descriptive analyses but excluded from gender-adjusted regression models because of sparse cell sizes, limiting the ability to examine HPV vaccination patterns among gender-diverse students. The HPV stigma composite was limited to two items and should be interpreted as exploratory. Finally, because the study did not measure all theoretical constructs directly, the results should not be interpreted as definitive evidence for any single behavioral model.

Overall, these findings suggest that perceived family vaccination context is an important correlate of self-reported HPV vaccine completion among medical students. The results are consistent with a view of HPV vaccination behavior as shaped by both individual knowledge and perceived social context, even within a medically trained population. Future research should validate these findings in larger, multi-institutional samples; use more comprehensive measures of family norms, stigma, and theoretical constructs; verify vaccination status when possible; and examine whether trainees’ own vaccination experiences are associated with future HPV counseling behavior.

## 5. Conclusions

Perceived family vaccination context was the strongest and most consistent correlate of self-reported HPV vaccine completion among medical students in this sample. Female gender and higher HPV knowledge were also associated with vaccination status in the primary adjusted model, while HPV stigma showed a borderline inverse association; however, these findings were less precise and should be interpreted cautiously. The results suggest that personal vaccination behavior among future physicians may not be explained by knowledge or formal education alone, but may also reflect perceived family-level vaccination context, stigma, and the broader social environment in which vaccination is discussed and normalized. Because this study was cross-sectional and relied on self-reported measures, findings should be interpreted as associations rather than causal effects. HPV prevention efforts in medical education may benefit from addressing factual knowledge, vaccination-status verification, trainees’ own vaccination histories, perceived family and social norms, and comfort discussing HPV vaccination. Exploratory findings related to recommendation intention and intent to vaccinate require confirmation in larger studies.

## Figures and Tables

**Table 1:** Sample Characteristics and Univariate Summary of Key Study Variables (N = 230).

Characteristic	Level	Overall
Total sample		230
HPV vaccination status	Yes	189 (82.2%)
	No	17 (7.4%)
	Unsure	24 (10.4%)
Gender	Female	141 (61.3%)
	Male	84 (36.5%)
	Non-binary, transgender, prefer not to say	5 (2.2%)
Religion	Christianity	106 (46.1%)
	Islam	17 (7.4%)
	No religion/atheist	57 (24.8%)
	Other religion	36 (15.7%)
	Missing/prefer not to say	14 (6.1%)
Age range	19–22	8 (3.5%)
	23–26	168 (73.0%)
	27–30	43 (18.7%)
	31–34	9 (3.9%)
	35–38	2 (0.9%)
Year in medical school	M1	46 (20.0%)
	M2	57 (24.8%)
	M3	76 (33.0%)
	M4	51 (22.2%)
Any prior HPV education	Yes	185 (80.4%)
	No	39 (17.0%)
	Missing	6 (2.6%)
Family members of age are vaccinated	Strongly agree	96 (41.7%)
	Agree	58 (25.2%)
	Neither agree nor disagree	9 (3.9%)
	Disagree	14 (6.1%)
	Strongly disagree	4 (1.7%)
	Not sure	49 (21.3%)
HPV knowledge score, 22-item	N non-missing	230
	Mean (SD)	16.48 (4.13)
	Median [IQR]	18 [15, 19]
	Range	1 to 22
	KR-20 reliability	0.81
HPV stigma composite	N non-missing	213
	Mean (SD)	3.45 (0.97)
	Median [IQR]	3.50 [3.00, 4.00]
	Cronbach α	0.71

Note: Gender categories are reported using the original survey response options.

**Table 2:** Bivariate Associations With HPV Vaccination Status.

Characteristic	Level	Vaccinated Yes (*n* = 189)	No/Unsure (*n* = 41)	Test	*p* Value
Gender	Female	122 (86.5%)	19 (13.5%)	Chi-square	0.045
	Male	63 (75.0%)	21 (25.0%)		
	Non-binary, transgender, prefer not to say	4	1	Excluded from test	
Religion	Christianity	86 (81.1%)	20 (18.9%)	Chi-square	0.458
	Islam	12 (70.6%)	5 (29.4%)		
	No religion/atheist	49 (86.0%)	8 (14.0%)		
	Other religion	31 (86.1%)	5 (13.9%)		
Age	Median [IQR]	2.0 [2.0, 2.0]	2.0 [2.0, 3.0]	Mann–Whitney U	0.582
Medical school year	Median [IQR]	3.0 [2.0, 3.0]	3.0 [1.0, 3.0]	Mann–Whitney U	0.798
Family vaccination context	Median [IQR]	5.0 [4.0, 5.0]	3.5 [2.0, 4.0]	Mann–Whitney U	<0.001
Any prior HPV education	Yes	158 (84.9%)	27 (15.1%)	Chi-square	0.068
	No	28 (71.8%)	11 (28.2%)		
HPV knowledge score, 22-item	Median [IQR]	18 [15, 19]	16 [13, 18]	Mann–Whitney U	0.006
HPV stigma composite	Median [IQR]	3.50 [3.00, 4.00]	4.00 [3.00, 4.00]	Mann–Whitney U	0.574

Note: Percentages are row percentages for categorical variables. Bivariate comparisons used complete available data for each variable. Gender test compared female and male respondents only; respondents reporting non-binary, transgender, or prefer-not-to-say gender identities were retained descriptively but excluded from the chi-square test because of sparse cell sizes. Ordinal and continuous variables were compared using Mann–Whitney U tests. The primary outcome coded fully vaccinated respondents as vaccinated and combined not fully vaccinated and unsure respondents as the comparison group. Gender categories are reported using the original survey response options.

**Table 3:** Multivariable Logistic Regression of HPV Vaccination Status.

Model	Predictor	OR (95% CI)	*p* Value
Primary adjusted model	Family vaccination context, per 1-step increase	3.95 (2.11–7.42)	<0.001
Primary adjusted model	Female vs. male	3.80 (1.01–14.29)	0.048
Primary adjusted model	HPV stigma composite, per 1-point increase	0.53 (0.26–1.07)	0.076
Primary adjusted model	HPV knowledge score, per 1-point increase	1.26 (1.03–1.53)	0.024
Primary adjusted model	Any prior HPV education, yes vs. no	4.91 (0.57–42.10)	0.147
Primary adjusted model	Religion: Islam vs. Christianity	2.07 (0.06–68.31)	0.684
Primary adjusted model	Religion: No religion/atheist vs. Christianity	1.44 (0.31–6.82)	0.643
Primary adjusted model	Religion: Other religion vs. Christianity	0.47 (0.09–2.55)	0.380
Primary adjusted model	Age, per category increase	0.97 (0.38–2.47)	0.944
Primary adjusted model	Medical school year, per year increase	0.51 (0.20–1.25)	0.142
Sensitivity model, yes vs. no only	Family vaccination context, per 1-step increase	7.70 (2.45–24.26)	<0.001
Sensitivity model, yes vs. no only	Female vs. Male	5.38 (0.58–50.18)	0.140
Sensitivity model, yes vs. no only	HPV stigma composite, per 1-point increase	0.22 (0.05–1.05)	0.057
Sensitivity model, yes vs. no only	HPV knowledge score, per 1-point increase	1.21 (0.84–1.75)	0.304
Sensitivity model, yes vs. no only	Any prior HPV education, yes vs. no	11.86 (0.62–226.04)	0.100
Sensitivity model, yes vs. no only	Religion: non-Christian vs. Christian	2.71 (0.35–21.09)	0.341
Sensitivity model, yes vs. no only	Age, per category increase	0.81 (0.20–3.38)	0.775
Sensitivity model, yes vs. no only	Medical school year, per year increase	0.49 (0.10–2.34)	0.371

Note: The primary adjusted model used complete-case binary logistic regression, *n* = 165. The primary outcome compared students who reported being fully vaccinated against HPV with students who reported being not fully vaccinated or unsure. The sensitivity model excluded respondents who were unsure of their HPV vaccination status and compared yes versus no only, *n* = 156. In the sensitivity model, religion was collapsed to Christian versus non-Christian because of sparse unvaccinated cells. Reference categories were male for gender and Christianity for religion. The family vaccination item treated “not sure” responses as missing. ORs greater than 1 indicate higher odds of being fully vaccinated. Gender categories are reported using the original survey response options.

## Data Availability

The data presented in this study are not publicly available because the dataset consists of student survey responses from a single medical school and includes combinations of demographic, educational, and attitudinal variables that could increase re-identification risk in a small source population. Although responses were collected anonymously and direct identifiers were not included in the analytic dataset, de-identified data may be available from the corresponding author upon reasonable request, subject to institutional approvals and privacy protections.

## Supplementary Materials

The following supporting information can be downloaded at: https://www.mdpi.com/article/10.3390/vaccines14070569/s1, File S1: Survey instrument used to assess HPV vaccination status, HPV knowledge, HPV stigma, family vaccination context, prior HPV education, recommendation intention, and demographic characteristics among medical students.
