# Supplementary material for: Family Vaccination Context Predicts HPV Vaccine Uptake Among Medical Students
Source: Vaccines (Basel). 2026 Jun 28;14(7):569. doi: 10.3390/vaccines14070569 (PMC13417496; doi:10.3390/vaccines14070569)
Supplement: Supplementary file 1 [file vaccines-14-00569-s001.zip › File S1 HPV Project.pdf]

HPV Project

Start of Block: Participant Information

Thank you for filling out this survey. We are two fellow WSU medical students that are conducting research on HPV-related cancer and vaccination.

This survey will ask you a series of questions on HPV-related morbidities and vaccination. You will then be presented with an infographic on HPV. After viewing the infographic, you will be again presented with the same questions in this survey. This survey will take approximately 10-15 minutes to complete. Please answer the questions to the best of your ability.

The first 200 participants that complete the entire survey will be rewarded with a $5 Amazon gift card sent to their Wayne State email!

No identifying information will be used in the results of this study.

If you have any questions regarding this study, please contact Akarsh Lal (akarshl@wayne.edu) or Laila Al-Jerdi (laila.al-jerdi@med.wayne.edu).

Please enter your Wayne email:

____

What year of medical school are you currently in?

- ○ M1 (1)
- ○ M2 (2)
- ○ M3 (3)
- ○ M4 (4)

What is your gender?

- ◯ Male (1)
- ◯ Female (2)
- ◯ Non-binary (3)
- ◯ Transgender male/man (4)
- ◯ Transgender female/woman (5)
- ◯ Genderqueer/Genderfluid (6)
- ◯ Agender (7)
- ◯ Other (8) ____
- ◯ Prefer not to say (9)

Which age range do you fall under?

- ◯ 19-22 (1)
- ◯ 23-26 (2)
- ◯ 27-30 (3)
- ◯ 31-34 (4)
- ◯ 35-38 (5)
- ◯ 39-42 (6)
- ◯ 43-46 (7)
- ◯ 47-50 (8)
- ◯ Other (9) ____

What is your religious affiliation?

- ○ Christianity (1)
- ○ Islam (2)
- ○ Judaism (3)
- ○ Hinduism (4)
- ○ Buddhism (5)
- ○ No religion/Atheist (6)
- ○ Other (please specify): (7) ____
- ○ Prefer not to say (8)

Have you had formal instruction (faculty lectures, Sketchy, Boards and Beyond, Pathoma, etc) regarding HPV in medical school yet?

- ○ Yes (1)
- ○ No (2)
- ○ Unsure (3)

End of Block: Participant Information

Start of Block: HPV and its morbidities

The following questions will ask you about HPV biology. Please answer each to the best of your ability.

Q1 HPV is a dsDNA virus.

- ○ Yes (1)
- ○ No (2)
- ○ Not sure (3)

Q2 Skin-to-skin contact of the genital area can spread HPV infection.

- ○ Yes (1)
- ○ No (2)
- ○ Not sure (3)

Q3 High-risk HPV infections are often asymptomatic.

- ○ Yes (1)
- ○ No (2)
- ○ Not sure (3)

Q4 HPV 6 and HPV 11 are low-risk HPV types.

- ○ Yes (1)
- ○ No (2)
- ○ Not sure (3)

Q5 HPV 16, 18, 31, and 33 are high-risk HPV types.

- ○ Yes (1)
- ○ No (2)
- ○ Not sure (3)

Q6 High-risk HPV can cause:

	True (1)	False (2)	Not sure (3)
Oropharyngeal cancer (1)	○	○	○
Cervical cancer (2)	○	○	○
Anal cancer (3)	○	○	○
Lung cancer (4)	○	○	○
Vaginal cancer (5)	○	○	○
Pancreatic cancer (6)	○	○	○
Penile cancer (7)	○	○	○
Vulvar cancer (8)	○	○	○

Q7 Please identify which trend on the graph below correlates with cervical and oropharyngeal cancer.
Please select all choices that are correct.

- ☐ Solid line is cervical cancer (1)
- ☐ Solid line is oropharyngeal cancer (2)
- ☐ Dashed line is cervical cancer (3)
- ☐ Dashed line is oropharyngeal cancer (4)

Sources: CDC's National Program of Cancer Registries; National Cancer Institute's Surveillance, Epidemiology, and End Results program.

Q8 HPV-related oropharyngeal cancers are often associated with tumors found in the base of the tongue and tonsils.

- ○ Strongly agree (1)
- ○ Agree (2)
- ○ Neither agree nor disagree (3)
- ○ Disagree (4)
- ○ Strongly disagree (5)
- ○ Not sure (6)

Q9 HPV-related oropharyngeal cancers often go undiagnosed due to a lack of symptoms in early stages.

- ○ Strongly agree (1)
- ○ Agree (2)
- ○ Neither agree nor disagree (3)
- ○ Disagree (4)
- ○ Strongly disagree (5)
- ○ Not sure (6)

Q10 The latency period between an oral HPV infection and the development of oropharyngeal cancer can span several decades.

- ○ Strongly agree (1)
- ○ Agree (2)
- ○ Neither agree nor disagree (3)
- ○ Disagree (4)
- ○ Strongly disagree (5)
- ○ Not sure (6)

Q11 High-risk HPV infections can avoid clearance by the body and enter a latent period.

- ○ Strongly agree (1)
- ○ Agree (2)
- ○ Neither agree nor disagree (3)
- ○ Disagree (4)
- ○ Strongly disagree (5)
- ○ Not sure (6)

End of Block: HPV and its morbidities

Start of Block: HPV Vaccination

The following questions are regarding HPV Vaccination. Please answer to the best of your ability.

Q12 HPV vaccination is recommended at age 11-12 for routine vaccination.

- ○ Strongly agree (1)
- ○ Agree (2)
- ○ Neither agree nor disagree (3)
- ○ Disagree (4)
- ○ Strongly disagree (5)
- ○ Not sure (6)

Q13 Two doses are recommended for immunocompromised persons.

- Strongly agree (1)
- Agree (2)
- Neither agree nor disagree (3)
- Disagree (4)
- Strongly disagree (5)
- Not sure (6)

Q14 HPV vaccination should be discussed with most adults over the age of 26.

- Strongly agree (1)
- Agree (2)
- Neither agree nor disagree (3)
- Disagree (4)
- Strongly disagree (5)
- Not sure (6)

Q15 The most common vaccine side effect is pain, redness, swelling at site of injection.

- ○ Strongly agree (1)
- ○ Agree (2)
- ○ Neither agree nor disagree (3)
- ○ Disagree (4)
- ○ Strongly disagree (5)
- ○ Not sure (6)

Q16 Vaccination CANNOT prevent cervical cancer deaths.

- ○ Strongly agree (1)
- ○ Agree (2)
- ○ Neither agree nor disagree (3)
- ○ Disagree (4)
- ○ Strongly disagree (5)
- ○ Not sure (6)

End of Block: HPV Vaccination

Start of Block: Attitudes and Perceptions

Q17 Are you currently fully vaccinated against HPV?

Full vaccination means you have received either:
 -Two doses if you started the vaccination series before age 15
OR
 -Three doses if you started the vaccination series between ages 15-26

- Yes (1)
- No (2)
- Unsure (3)

Q18 If appropriate, I will encourage HPV vaccination to my patients in the future as a physician.

- Strongly agree (1)
- Agree (2)
- Neither agree nor disagree (3)
- Disagree (4)
- Strongly disagree (5)
- Not sure (6)

Q19 Family members of age are vaccinated.

- Strongly agree (1)
- Agree (2)
- Neither agree nor disagree (3)
- Disagree (4)
- Strongly disagree (5)
- Not sure (6)

Display this question:

If Family members of age are vaccinated. = Strongly agree

Or Family members of age are vaccinated. = Agree

Please specify, ie, parents, siblings, cousins, male/female

____

____

____

____

____

Q20 In the next 3 months, I will encourage HPV vaccination with friends and family.

- Strongly agree (1)
- Agree (2)
- Neither agree nor disagree (3)
- Disagree (4)
- Strongly disagree (5)
- Not sure (6)

Q21 If I am not fully vaccinated for HPV, I plan to get fully vaccinated in the next 3 months.

- Strongly agree (1)
- Agree (2)
- Neither agree nor disagree (3)
- Disagree (4)
- Strongly disagree (5)
- Not sure (6)

Q22 Having HPV carries the same stigma as other sexually transmitted infections.

- Strongly agree (1)
- Agree (2)
- Neither agree nor disagree (3)
- Disagree (4)
- Strongly disagree (5)
- Not sure (6)

Q23 There is a stigma associated with telling family and friends that a person is diagnosed with HPV.

- ◯ Strongly agree (1)
- ◯ Agree (2)
- ◯ Neither agree nor disagree (3)
- ◯ Disagree (4)
- ◯ Strongly disagree (5)
- ◯ Not sure (6)

Q24 Medical schools should increase HPV-focused education to increase vaccination rates and HPV knowledge.

- ◯ Strongly agree (1)
- ◯ Agree (2)
- ◯ Neither agree nor disagree (3)
- ◯ Disagree (4)
- ◯ Strongly disagree (5)
- ◯ Not sure (6)

Q25 I have previously taken a course on HPV or extensively studied HPV outside of medical school.

- ○ Strongly agree (1)
- ○ Agree (2)
- ○ Neither agree nor disagree (3)
- ○ Disagree (4)
- ○ Strongly disagree (5)
- ○ Not sure (6)

End of Block: Attitudes and Perceptions

Start of Block: Block 4

Please take 4-5 minutes to view this infographic. After viewing the infographic, please move ahead to the next part of the survey to answer the post-infographic questions. These questions are the same as the questions you previously answered.

End of Block: Block 4

Start of Block: Post-Survey: HPV and its morbidities

Please answer the following questions after having reviewed the infographic.

Q26 HPV is a dsDNA virus.

- Strongly agree (1)
- Agree (2)
- Neither agree nor disagree (3)
- Disagree (4)
- Strongly disagree (5)
- Not sure (6)

Q27 HPV targets and disrupts cell growth and division of epithelial cells in areas like the throat, tonsils, and cervix.

- Strongly agree (1)
- Agree (2)
- Neither agree nor disagree (3)
- Disagree (4)
- Strongly disagree (5)
- Not sure (6)

Q28 Skin-to-skin contact of the genital area can spread HPV infection.

- Strongly agree (1)
- Agree (2)
- Neither agree nor disagree (3)
- Disagree (4)
- Strongly disagree (5)
- Not sure (6)

Q29 High-risk HPV infections are often asymptomatic.

- Strongly agree (1)
- Agree (2)
- Neither agree nor disagree (3)
- Disagree (4)
- Strongly disagree (5)
- Not sure (6)

Q30 HPV 6 and HPV 11 are low-risk HPV types.

- Strongly agree (1)
- Agree (2)
- Neither agree nor disagree (3)
- Disagree (4)
- Strongly disagree (5)
- Not sure (6)

Q31 HPV 16, 18, 31, and 33 are high-risk HPV types.

- Strongly agree (1)
- Agree (2)
- Neither agree nor disagree (3)
- Disagree (4)
- Strongly disagree (5)
- Not sure (6)

Q32 High-risk HPV can cause:

	True (1)	False (2)	Not sure (3)
Oropharyngeal cancer (1)	○	○	○
Cervical cancer (2)	○	○	○
Anal cancer (3)	○	○	○
Lung cancer (4)	○	○	○
Vaginal cancer (5)	○	○	○
Pancreatic cancer (6)	○	○	○
Penile cancer (7)	○	○	○
Vulvar cancer (8)	○	○	○

Q33 Please identify which trend on the graph below correlates with cervical and oropharyngeal cancer.
Please select all choices that are correct.

- ▢ Solid line is cervical cancer (1)
- ▢ Solid line is oropharyngeal cancer (2)
- ▢ Dashed line is cervical cancer (3)
- ▢ Dashed line is oropharyngeal cancer (4)

Sources: CDC's National Program of Cancer Registries; National Cancer Institute's Surveillance, Epidemiology, and End Results program.

Q34 The latency period between an oral HPV infection and the development of oropharyngeal cancer can span several decades.

- Strongly agree (1)
- Agree (2)
- Neither agree nor disagree (3)
- Disagree (4)
- Strongly disagree (5)
- Not sure (6)

Q35 High-risk HPV infections can avoid clearance by the body and enter a latent period.

- Strongly agree (1)
- Agree (2)
- Neither agree nor disagree (3)
- Disagree (4)
- Strongly disagree (5)
- Not sure (6)

End of Block: Post-Survey: HPV and its morbidities

Start of Block: Post-Survey HPV Vaccination

Q36 HPV vaccination is recommended at age 11-12 for routine vaccination.

- Strongly agree (1)
- Agree (2)
- Neither agree nor disagree (3)
- Disagree (4)
- Strongly disagree (5)
- Not sure (6)

Q37 Two doses are recommended for immunocompromised persons.

- Strongly agree (1)
- Agree (2)
- Neither agree nor disagree (3)
- Disagree (4)
- Strongly disagree (5)
- Not sure (6)

Q38 HPV vaccination should be discussed with most adults over the age of 26.

- ○ Strongly agree (1)
- ○ Agree (2)
- ○ Neither agree nor disagree (3)
- ○ Disagree (4)
- ○ Strongly disagree (5)
- ○ Not sure (6)

Q39 The most common vaccine side effect is pain, redness, swelling at site of injection.

- ○ Strongly agree (1)
- ○ Agree (2)
- ○ Neither agree nor disagree (3)
- ○ Disagree (4)
- ○ Strongly disagree (5)
- ○ Not sure (6)

Q40 Vaccination CANNOT prevent cervical cancer deaths.

- Strongly agree (1)
- Agree (2)
- Neither agree nor disagree (3)
- Disagree (4)
- Strongly disagree (5)
- Not sure (6)

End of Block: Post-Survey HPV Vaccination

Start of Block: Post-Survey Attitudes and Perceptions

Q41 Are you currently fully vaccinated against HPV?

Full vaccination means you have received either:
 -Two doses if you started the vaccination series before age 15
OR
 -Three doses if you started the vaccination series between ages 15-26

- Yes (1)
- No (2)
- Unsure (3)

Q42 My understanding of HPV increased after participating in this study.

- ○ Strongly agree (1)
- ○ Agree (2)
- ○ Neither agree nor disagree (3)
- ○ Disagree (4)
- ○ Strongly disagree (5)
- ○ Not sure (6)

Q43 If appropriate, I will encourage HPV vaccination to my patients in the future.

- ○ Strongly agree (1)
- ○ Agree (2)
- ○ Neither agree nor disagree (3)
- ○ Disagree (4)
- ○ Strongly disagree (5)
- ○ Not sure (6)

Q44 My family members of age are vaccinated.

- ◯ Strongly agree (1)
- ◯ Agree (2)
- ◯ Neither agree nor disagree (3)
- ◯ Disagree (4)
- ◯ Strongly disagree (5)
- ◯ Not sure (6)

Display this question:

If My family members of age are vaccinated. = Strongly agree

Or My family members of age are vaccinated. = Agree

Please specify, ie, parents, siblings, cousins, male/female

____

____

____

____

____

Q45 In the next 3 months, I will encourage HPV vaccination with friends and family.

- ○ Strongly agree (1)
- ○ Agree (2)
- ○ Neither agree nor disagree (3)
- ○ Disagree (4)
- ○ Strongly disagree (5)
- ○ Not sure (6)

Q46 If I am not fully vaccinated for HPV, I plan to get fully vaccinated in the next 3 months.

- ○ Strongly agree (1)
- ○ Agree (2)
- ○ Neither agree nor disagree (3)
- ○ Disagree (4)
- ○ Strongly disagree (5)
- ○ Not sure (6)

Q47 Having HPV carries the same stigma as other sexually transmitted infections.

- ○ Strongly agree (1)
- ○ Agree (2)
- ○ Neither agree nor disagree (3)
- ○ Disagree (4)
- ○ Strongly disagree (5)
- ○ Not sure (6)

Q48 Please describe factors (if any) that prevent you from discussing HPV vaccination in your community? If there are none, please write NA for not applicable.

____

Q49 There is a stigma associated with telling family and friends that a person is diagnosed with HPV.

- ○ Strongly agree (1)
- ○ Agree (2)
- ○ Neither agree nor disagree (3)
- ○ Disagree (4)
- ○ Strongly disagree (5)
- ○ Not sure (6)

Q50 Medical schools should increase HPV-focused education to increase vaccination rates and HPV knowledge.

- Strongly agree (1)
- Agree (2)
- Neither agree nor disagree (3)
- Disagree (4)
- Strongly disagree (5)
- Not sure (6)

Q51 I have previously taken a course or extensively studied HPV outside of medical school.

- Strongly agree (1)
- Agree (2)
- Neither agree nor disagree (3)
- Disagree (4)
- Strongly disagree (5)
- Not sure (6)

End of Block: Post-Survey Attitudes and Perceptions
