# Supplementary figures and images for: Family Vaccination Context Predicts HPV Vaccine Uptake Among Medical Students
Source: Vaccines (Basel). 2026 Jun 28;14(7):569. doi: 10.3390/vaccines14070569 (PMC13417496; doi:10.3390/vaccines14070569)

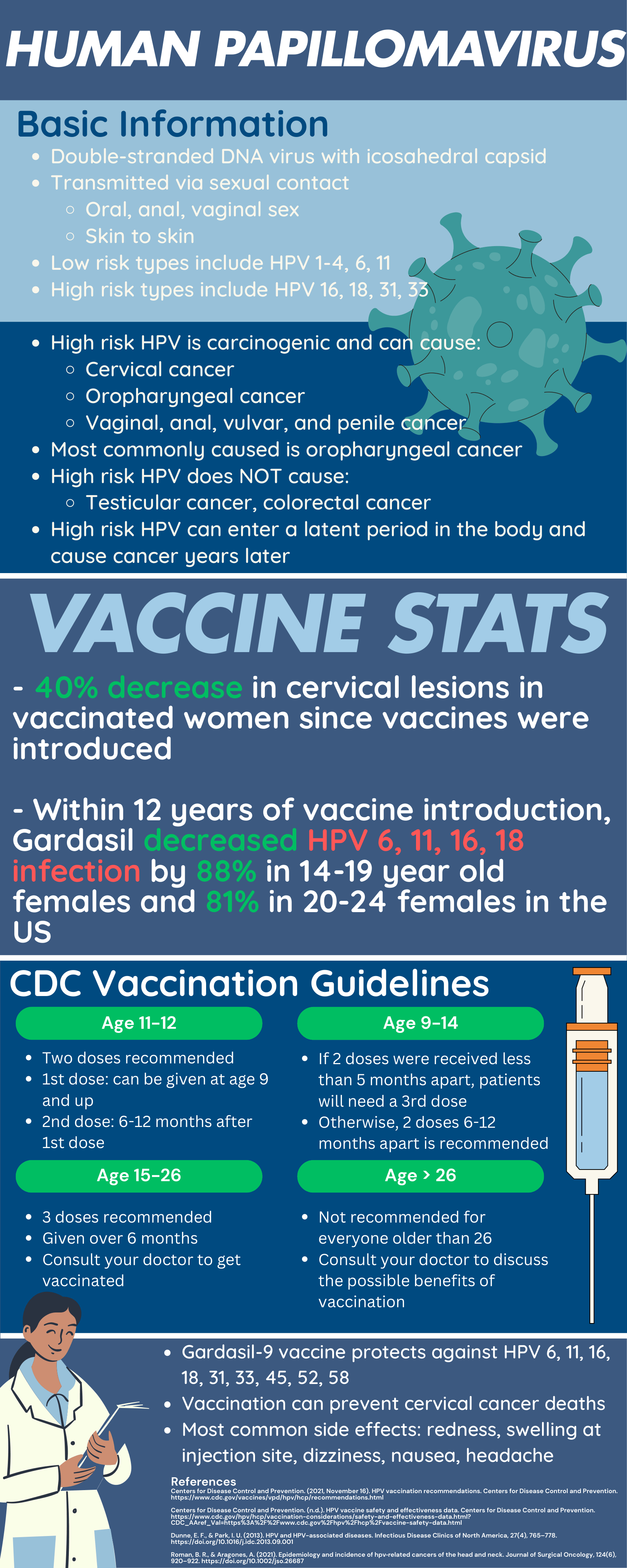

Supplement: Supplementary file 1 [file vaccines-14-00569-s001.zip › HPV Infographic.png]
